# Stem of *Sorbus commixta* Hedl. Extract Inhibits Cartilage Degradation and Arthritic Pain in Experimental Model via Anti-Inflammatory Activity

**DOI:** 10.3390/nu15173774

**Published:** 2023-08-29

**Authors:** Hee-Geun Jo, Chae Yun Baek, Donghwan Kim, Donghun Lee, Ho Sueb Song

**Affiliations:** 1 Department of Herbal Pharmacology, College of Korean Medicine, Gachon University, 1342 Seongnamdae-ro, Sujeong-gu, Seongnam-si 13120, Republic of Korea; jho3366@hanmail.net (H.-G.J.); cyning20@gachon.ac.kr (C.Y.B.); 2 Naturalis Inc. 6, Daewangpangyo-ro, Bundang-gu, Seongnam-si 13549, Republic of Korea; 3 Department of Acupuncture & Moxibustion Medicine, College of Korean Medicine, Gachon University, 1342 Seongnamdae-ro, Sujeong-gu, Seongnam-si 13120, Republic of Korea

**Keywords:** East Asian herbal medicine, osteoarthritis, sorbus commixta, anti-inflammatory, network pharmacology, cartilage degradation

## Abstract

Osteoarthritis (OA) is a widespread joint disease that affects millions of people worldwide. Conventional treatments for OA, including non-steroidal anti-inflammatory drugs (NSAIDs) and steroids, have a risk of various adverse events, including liver, gastrointestinal, cardiovascular, and kidney disease, which are unsatisfactory in their effectiveness. In this study, *Sorbus commixta* Hedl. Stem extracts (SCE) were evaluated in animal models as potential inhibitors for the progression of OA. *Sorbus commixta* Hedl., which was found to have substantial anti-inflammatory and antioxidant activities in earlier investigations, has shown potential as a candidate for OA treatment. To mimic human OA symptoms, male rats were injected using sodium iodoacetate (MIA) in their knee joints. SCE significantly reduced MIA-induced weight-bearing loss in rats after the MIA injection and alleviated cartilage degradation and subchondral bone injury caused by MIA. In addition, SCE administration reduced levels of TNF-α and IL-1β such as pro-inflammatory cytokines in serum, as well as the levels of matrix metalloproteinases (MMPs) such as MMP-1, -3, -8 and -13 in the joint cartilage. SCE significantly inhibited the writhing responses in acetic acid-administered mice and was used to quantify pain. In lipopolysaccharide (LPS)-activated RAW264.7, SCE suppressed NO production and reduced the expression of TNF-α, PGE2, IL-6, IL-1β, MMP1, MMP3, MMP8, and MMP-13. Our study showed that SCE alleviated inflammation and cartilage degradation in arthritis through its anti-inflammatory activities on multiple targets.

## 1. Introduction

Osteoarthritis (OA) is one of the most common diseases in modern society, affecting more than five million people worldwide [1]. The major challenge associated with this disease is the irreversible course of chronic pain and progressive joint destruction, leaving individuals with the burden of reduced daily function and quality of life [2,3]. Furthermore, epidemiological studies covering most countries worldwide have reported an annual increase in OA of 7.8% between 1990 and 2019 [4]. Consequently, the socioeconomic costs of the disease have become an increasingly important issue in medical research [5]. OA is a degenerative disease primarily associated with accumulated damage to musculoskeletal structures owing to mechanical stress. Therefore, temporary symptomatic relief with paracetamol and nonsteroidal anti-inflammatory drugs (NSAIDs) in combination with exercise has been recommended as the primary management approach [6,7]. However, these interventions have small effect sizes and have not been shown to inhibit the progressive worsening of OA, which is the most important long-term goal [2,8]. In addition, safety concerns related to the increased risk of serious hepatic, gastrointestinal, cardiovascular, and renal adverse events associated with current medications are among the unmet medical needs of patients with OA [6,9,10].

Recently, there has been substantial evidence that low-grade intra-articular synovial inflammation directly contributes to pain and radiographic progression of OA [11]. Thus, targeting anti-inflammatory pharmacology, which involves inhibiting the activity of pro-inflammatory mediators secreted by the synovium and cartilage in the affected joint, holds promise for achieving therapeutic goals such as pain relief and inhibiting structural damage in OA [12,13]. This advanced understanding of the pathophysiology of OA, coupled with a variety of newly identified targets and pathways, is driving the development of disease-modifying osteoarthritis drugs (DMOADs) as a new class of interventions that can reduce the disease burden and delay the natural history of OA, including progressive joint destruction [14]. none of the DMOAD candidates have been approved for market use due to a lack of evidence of long-term efficacy and safety [15]. The difficulty in developing such therapies stems from the fact that OA is not a disease with a single phenotype or pathological pathway [3,16]. Therefore, it is necessary to explore drug discovery strategies that can simultaneously modulate the multiple therapeutic targets and pathophysiologies involved in the pathology of OA as a prerequisite for developing drugs that can meaningfully reduce symptoms and joint destruction while ensuring safety during long-term administration.

Natural products are a group of safe candidates already tested in humans for various pain conditions, and the multi-ingredient and multi-pharmacological effects of natural products are believed to be well-positioned to meet this demand [17,18,19,20,21,22]. In particular, East Asian herbal medicines have accumulated a large body of scientific evidence on both the mechanisms of clinical arthritis symptom improvement and inhibition of inflammatory pathology based on their long-term historical use in the region and can therefore be considered a high quality data pool for DMOAD discovery [23,24,25,26,27,28,29]. Among them, *Sorbus commixta* Hedl., which has been reported to have significant anti-inflammatory and antioxidant activities in previous studies, has shown promise as a candidate for the treatment of OA [30,31]. More specifically, the stem and bark of this medicinal plant have been used in Korea to treat various inflammatory diseases, and individual active compounds have been reported to have negative regulation of the NF-κB pathway and inhibitory effects on osteoclast differentiation and bone resorption [32,33]. These studies and experiences suggest that the stems of *Sorbus commixta* Hedl. is a promising material with anti-osteoarthritic activity. However, there have been no specific investigations of its potential. Hence, in order to explore the potential of this herbal material as a DMOAD, experimental observations are needed to determine whether it can inhibit progressive joint destructive pathology through anti-inflammatory mechanisms and not just symptomatic improvement activity. Furthermore, as the reported antioxidant properties of the materials are likely to be involved in the protection of cartilage damage, the identification of their effects and targets of action should also be an essential research goal [34,35].

Therefore, in the present study we evaluated the effects of *Sorbus commixta* Hedl. stem extract (SCE) on certain biochemical parameters, inflammatory status, and the morphological features of monosodium iodoacetate (MIA)-induced knee OA in rats. Additionally, we cross-validated the analgesic effect using an acetic acid-induced mouse writhing model and explored the possible mechanism of action by combining the pathophysiology of OA with in vitro experiments on various inflammatory cytokines and catabolic markers.

## 2. Materials and Methods

### 2.1. Material Preparation

The stems of *Sorbus commixta* Hedl. were acquired from Yaksudang Pharma Co., Ltd. (Seoul, Republic of Korea). The voucher specimen was committed to Professor Donghun Lee in Department of Herbal Pharmacology at Gachon University (2009150004). Dried stem of *Sorbus commixta* Hedl. was extracted under reflux at 85 °C for 3 h with 30% ethanol. The filtered and concentrated extracts were lyophilized. The yield was 4.97%. The extract was then lyophilized at −80 °C.

### 2.2. Data Sources and Search Strategy High Performance Liquid Chromatography (HPLC) Analysis

The HPLC carried out a 1100 series HPLC system (Agilent Technologies, Inc., Santa Clara, CA, USA) was used to perform the chromatographic analysis of SCE. Chromatographic separation was performed at 30 °C on a Zorbax Eclipse XDB 5 µm C18 (4.6 × 250 mm) column. A sample of 10 mg was diluted in 1 mL of ethanol and sonicated for 10 min. A 0.2 μm syringe filter (Agilent, Santa Clara, CA, USA) was used to filter the samples. The mobile phase comprised 2% acetic acid (A) and 0.5% acetic acid with 50% acetonitrile (B). The column was eluted as follows: 0–10 min, 0–0%; 10–140 min, 0–50%; 140–180 min, 50–70%; 180–190 min, 70–70%; 190–195 min, 70–0%; 195–200 min, 0–0% solvent B at the flow rate of 1.0 mL/min. Outflow was recorded at 327 nm with the injection volume of 10 μL. The experiments were carried out in triplicates.

### 2.3. Animal

DBL Co., Ltd. (DBL, Incheon, Republic of Korea) provided 6-week male ICR mice and 5-week male Sprague-Dawley (SD) rats. The Gachon University Center of Animal Care and Use approved all experimental protocols (GIACUC-R2020028). The animal room was kept at a temperature of 22 ± 2 °C, humidity of 55 ± 10%, and a light condition of 12 h. Every animal was freely provided with food and water. All animals were allowed to adapt for 7 days.

### 2.4. OA Induction and Diet Preparation

The rats were allocated randomly into five groups (each group, *n* = 9) and allowed to adapt for seven days before treatment. The five groups were as follows: the sham, control, indomethacin, SCE 100 mg/kg, and SCE 300 mg/kg. To induce OA, the rats were anesthetized with 2% isofluorane and a combination of nitrous oxide and oxygen before being injected intra-articularly with 40 mg/mL MIA (Sigma, St. Louis, MO, USA). The treatments were conducted in the following manner. The AIN-93G diet was fed to the sham and control groups. The indomethacin group received indomethacin (3 mg/kg) included into the AIN-93G diet, while the SCE groups received the AIN-93G diet with SCE (100 and 300 mg/kg). The rats were sacrificed on day 24 after MIA injection and had blood and right knee joint cartilage tissue collected.

### 2.5. Serum Measurement of OA Induction in MIA Rats

After 30 min at RT, the blood samples were centrifuged at 2688× *g* for 10 min at 4 °C to separate the serum. The separated serum was kept at −70 °C. For cytokine measurements in serum, a multiplex assay was assessed using IL-1β and TNF-α at PremixedMultiAnalyte Kit (R&D Systems Inc., Minneapolis, MN, USA), and the findings were evaluated with Luminex MAGPIX instruments (Luminex Corp., Austin, TX, USA). All multiplex assays were carried out in accordance with the manufacturer’s guidelines.

### 2.6. Weight-Bearing Analysis

Weight-bearing was recorded using an IITC Life Science 600 Incapacitance Meter Tester (IITC-Life Science Inc., Los Angeles, CA, USA) from the first day of MIA induction to 24 days after OA induction, and the strength delivered to each leg was calculated as the average over 10 s. The following equation calculates the weight arrangement of the right hind leg.

$$\mathrm{weight}\;\mathrm{bearing}\;\mathrm{ratio}\left(\%\right)=\frac{\mathrm{weight}\;\mathrm{on}\;\mathrm{right}\;\mathrm{hind}\;\mathrm{limb}}{\mathrm{weight}\;\mathrm{on}\;\mathrm{left}\;\mathrm{and}\;\mathrm{right}\;\mathrm{hind}\;\mathrm{limb}}*100$$

### 2.7. Writhing Response Induced by Acetic Acid (AA)

The mice were allocated randomly into four groups (each group, *n* = 8) and samples were treated with control (distilled water), SCE (200 and 600 mg/kg), or ibuprofen (200 mg/kg; Sigma, USA) 30 min before AA injection. Acetic acid (10 mL/kg, 0.7%) was injected intraperitoneally 10 min before recording and was counted 10 min later. The writhing response consisted of the abdominal wall tightness and pelvic rotation, followed by the hind legs swelling.

### 2.8. Cell Culture

RAW264.7 cell lines were bought at the American Type Culture Collection (ATCC Inc., Manassas, VA, USA). The cells were cultured at 37 °C incubator with 5% CO_2_. The culture medium was composed of 100 U/mL penicillin-streptomycin, 10% FBS (WELGENE Inc., Gyeongsangbukdo, Republic of Korea) in DMEM medium.

### 2.9. Generation of NO and Cell Toxicity Evaluation

The RAW264.7 cells were grown on the 96 well cell culture plates at a cell density of 1 × 10^4^ cells/well. The cells were cultured with a medium containing SCE (10–1000 g/mL) and lipopolysaccharide (LPS, 500 ng/mL) during a 24 h period. The culture supernatant liquid was mixed with Greiss reagent (1:1), and the absorbance was measured at 540 nm. Mitochondrial reduction activity (MRA) was evaluated using the Ez-Cytox reagent (DoGenBio, Seoul, Republic of Korea) according to the manufacturer’s guidelines.

### 2.10. Quantitative Real-Time Polymerase Chain Reaction (qRT-PCR)

RNAs were extracted from OA-induced cartilage tissues and LPS-activated RAW264.7 cells using RNA Extraction Kit (TaKaRa Inc., Seoul, Republic of Korea) and then converted cDNA by PrimeScript™ cDNA Synthesis Kit (TaKaRa, Republic of Korea) following the manufacturer’s protocol. Expression of mRNA was quantified using the AccuPower^®^-2XGreenStar ^®^ qPCR Master Mix (Bioneer, Daejeon, Republic of Korea) and primers (Table 1 and Table 2). The primer sequences utilized were as follows:

### 2.11. Western Blot

Total protein was isolated from the knee cartilage tissues and LPS-activated RAW264.7, using a homogenizer (NISSEI CORPERATION, Toyama, Japan) with RIPA lysis buffer (CST Inc., Peachtree City, GA, USA) and Protease Inhibitor Cocktail with EDTA-free (Sigma, USA). Western blot assay was utilized to examine the protein expression of MMP-1, -3, -13, IL-6, TNF-α, IL-1β, iNOS, and GAPDH. The total protein (10 µg) from every sample was separated by SDS-PAGE gel and transferred to a PVDF membrane using a Semidry-Transfer (BioRad Lab., Inc., Hercules, CA, USA) for 1 h at 15 V. 1st antibodies (MMP-1, -3, -13, IL-6, TNF-α, IL-1β, iNOS, and GAPDH) incubated at the PVDF membranes at 4 °C for 24 h. The antibodies were obtained from Abcam Corp. (Waltham, MA, USA), CST, Inc., and Proteintech Group, Inc. (Rosemont, IL, USA). The membranes were cultured with a 2nd antibody for 1 h at RT and visualized by ECL Substrate (Bio-Rad Inc., USA) solution. Western blot images were analyzed using an Azure 280 instrument (Azure Biosystems, Dublin, CA, USA).

### 2.12. Statistics

Statistical analyses were conducted using GraphPad Prism9 (GraphPad Software, San Diego, CA, USA) with 1-way ANOVA and Dunnett’s post hoc test. Differences were considered statistically significant at *p* < 0.05, and measurements are shown as mean ± standard error of the mean.

## 3. Results

### 3.1. HPLC Analysis

**Figure 1:**
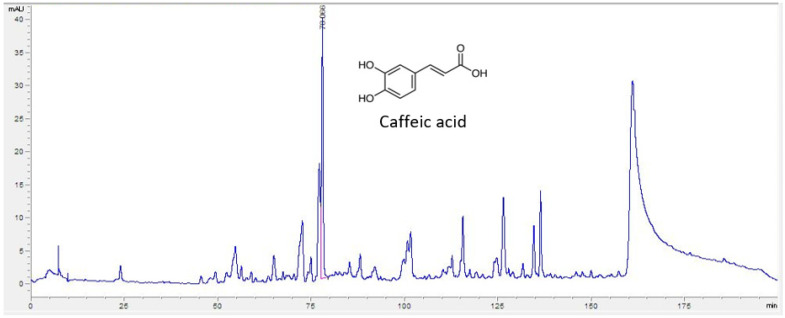
HPLC chromatogram of *Sorbus commixta* extract. *X*-axis; retention time; *Y*-axis; absorbance unit. The monitoring wavelength for caffeic acid was set at 327 nm.

The HPLC chromatogram and the constituent compound chemical structures, are shown in Figure 1. In this study, caffeic acid was detected in SCE by HPLC-UV. The extract contains 4.3 μg/g of caffeic acid.

### 3.2. Effects on Weight-Bearing Arrangement in OA-Induction MIA Rats

**Figure 2:**
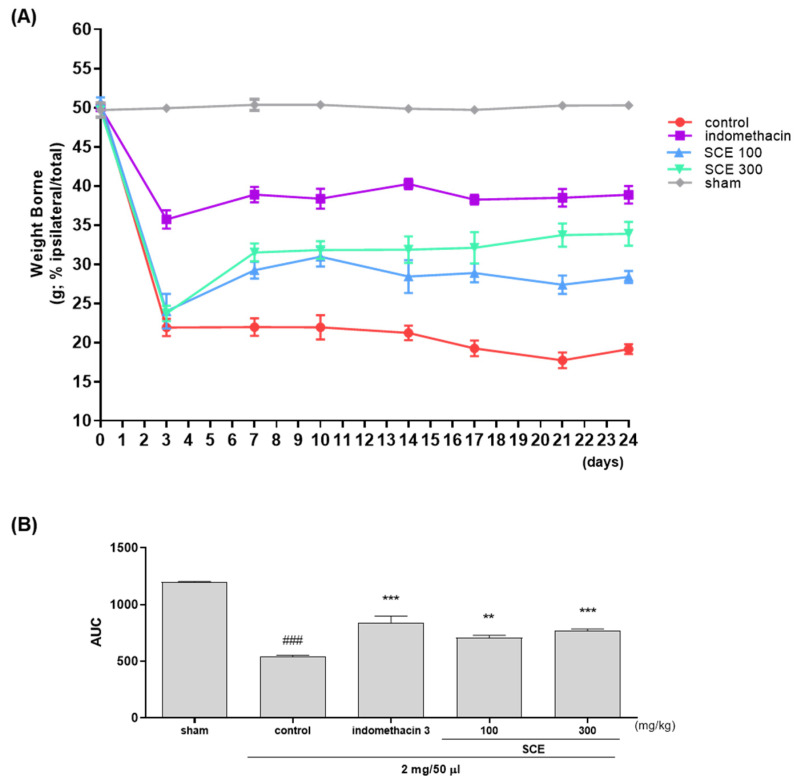
Effects of *Sorbus commixta* extract (SCE) on the weight-bearing arrangement of hind legs in OA induction MIA rats. (**A**) Weight-bearing arrangement of OA-induction MIA rats from 0 to 24 days and (**B**) The measurement of area under the curve (AUC) with an incapacitance meter. ** *p* < 0.05 vs. control, *** *p* < 0.001 vs. control and ### *p* < 0.001 vs. sham by one-way ANOVA, Dunnett’s test.

Weight-bearing arrangements were measured 24 days after OA induction using MIA. In contrast to the sham group, the weight-bearing arrangements in the control group were considerably altered on day three and persisted thereafter, as shown in Figure 2A. However, the SCE treatment significantly alleviated weight bearing in MIA rats. The weight-bearing increase of 300 mg/kg of SCE was equivalent to the indomethacin group (Figure 2B).

### 3.3. Effects on Knee Joint Damage in OA-Induction MIA Rats

**Figure 3:**
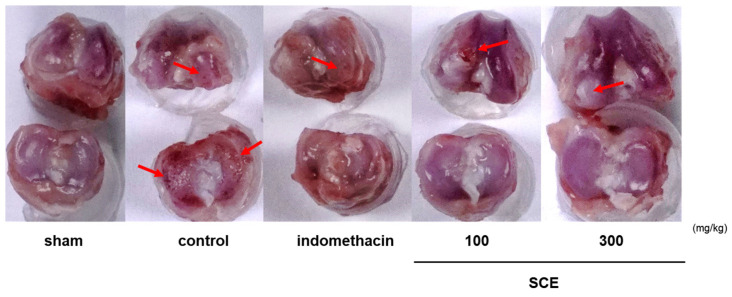
Photographs of the joint cartilages of rats with Monosodium iodoacetate (MIA)-induced osteoarthritis. OA-induction MIA rats were administered with 3 mg/kg indomethacin and 100 and 300 mg/kg *Sorbus commixta* extract. Arrows indicated the cartilage erosion spot.

A representative image of each experimental group’s knee joints indicates that SCE prevented cartilage degradation caused by the MIA injection. As shown in Figure 3, the knee joint cartilage of sham rats was glossy and smooth, but the cartilage of the control rats was less polished and rougher, with some areas damaged. The cartilage degradation caused by MIA was significantly reduced in rats treated with SCE or indomethacin. Notably, the recovery of cartilage degradation by SCE was comparable to that of indomethacin.

### 3.4. Effects on Inflammatory Cytokines in OA-Induction MIA Rats

**Figure 4:**
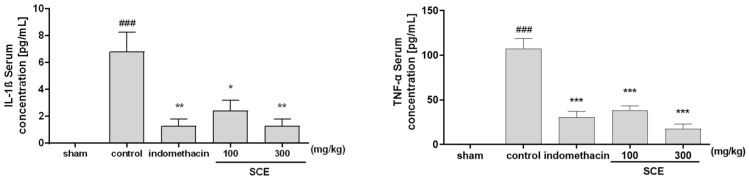
Inflammatory cytokine (IL-1β and TNF-α) levels in serum of OA-induction monosodium-iodoacetate (MIA) rats. Rats were administered with 100 and 300 mg/kg SCE for 24 days. * *p* < 0.01 vs. control, ** *p* < 0.05 vs. control, *** *p* < 0.001 vs. control, and ### *p* < 0.001 vs. sham by one-way ANOVA and Dunnett’s test.

Levels of IL-1β and TNF-β in the serum of experimental rats were measured. In a dose-dependent manner, the SCE-treated group identified a significant reduction in serum concentrations of IL-1β and TNF-α when contrasted with the control group. The 300 mg/kg SCE group decreased the IL-1β and TNF-α levels similar to the indomethacin-treated group (Figure 4).

### 3.5. Effect on Acetic Acid-Induced(AA) Writhing Responses

**Figure 5:**
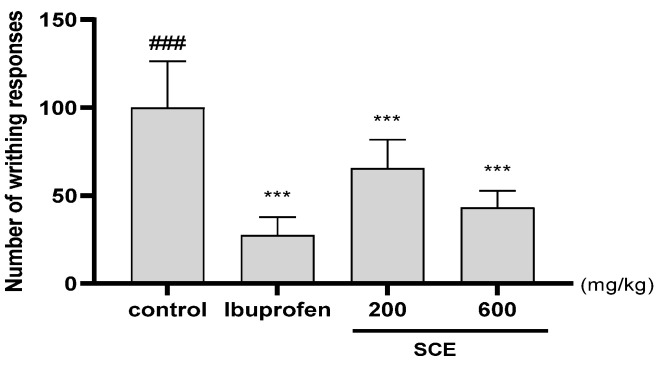
The number of writhing responses in ICR mice induced by AA. 30 min after oral administration, each mouse received an intraperitoneal injection of 0.7% AA before 10 min of counting. The number of mice was 8 per group; *** *p* < 0.001 vs. contro, and ### *p* < 0.001 vs. sham by one-way ANOVA and Dunnett’s test.

AA-induced writhing responses in mice were used to investigate the analgesic effects of SCE. The analgesic effect of SCE was observed in AA-induced writhing in mice by analyzing writhing responses. After 10 min, the average writhing response in the control group was 100. Compared to the control, SCE treatment reduced the number of writhing cells. These results demonstrate the analgesic effects of SCE (Figure 5).

### 3.6. Effects on Cytokines Responses in Cartilage Tissue

**Figure 6:**
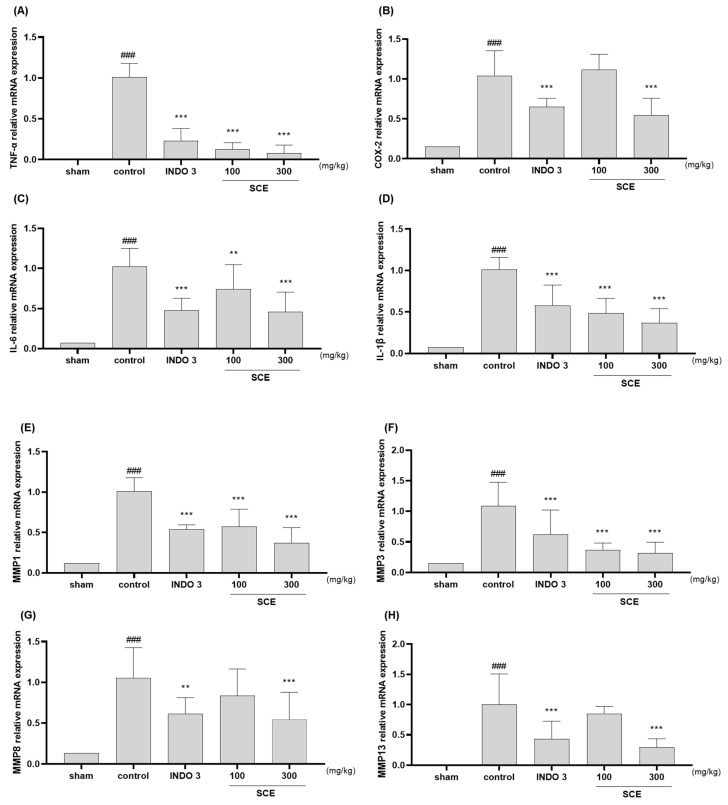

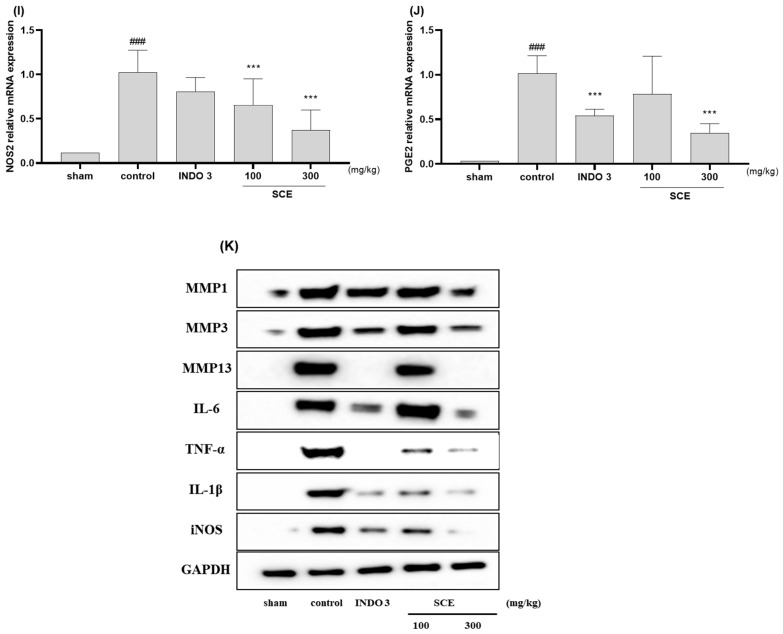
Changes in cytokines at cartilage tissue at 100 and 300 mg/kg SCE treatment. (**A**–**J**) mRNA expression of TNF-α, COX-2, IL-6, IL-1β, MMP-1, -3, -8, -13, NOS2, and PGE2 (Table 1) determined by qRT-PCR. (**K**) Protein expression of TNF-α, IL-6, IL-1β, MMP-1, -3, -8, -13, iNOS, and PGE2 analyzes with Western blot assay. ** *p* < 0.05 vs. control, *** *p* < 0.001 vs. control, and ### *p* < 0.001 vs. sham by one-way ANOVA and Dunnett’s test. NT: non-treated INDO 3: 3 mg/kg of indomethacin.

The analysis of TNF-α, COX-2, IL-6, IL-1β, MMP-1, -3, -8, -13, NOS2, and PGE2 mRNA levels (Figure 6A–J) in rats identified that the SCE treatment significantly decreased the knee joint cartilage tissue when compared to the control rats. The anti-inflammatory effect of SCE was dose-dependent, and 300 SCE showed effects similar to those of dexamethasone. Noticeably, 300 SCE rats had lower TNF-α, IL-1β, MMP-1, -3, -13, NOS2, and PGE2 levels than the indomethacin-treated group. Western blot examination also revealed that SCE inhibited TNF-α, IL-6, IL-1β, MMP-1, -3, -8, -13, iNOS, and PGE2 in the MIA rats.

### 3.7. Effects on Anti-Inflammatory in the LPS-Activated RAW264.7 Cells

**Figure 7:**
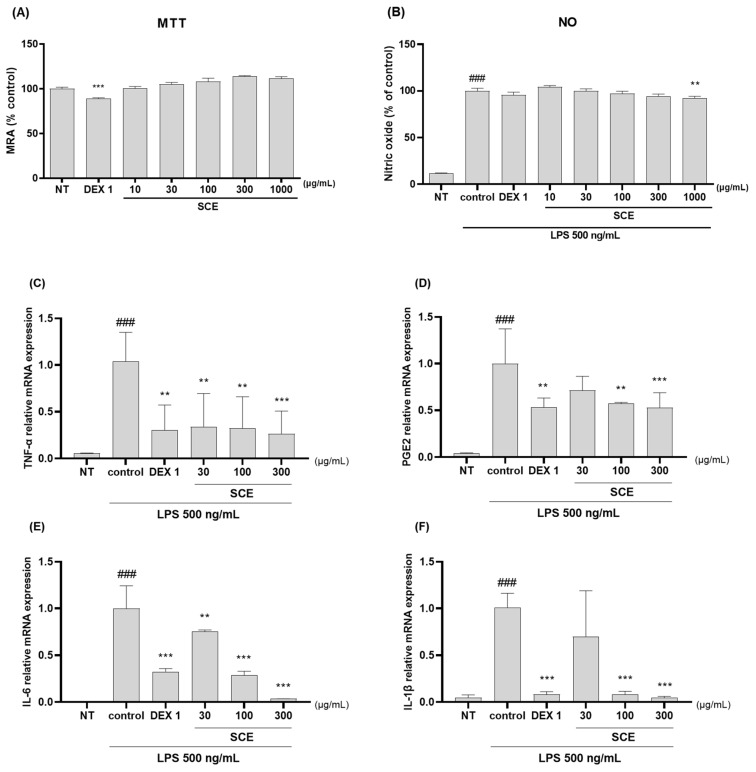

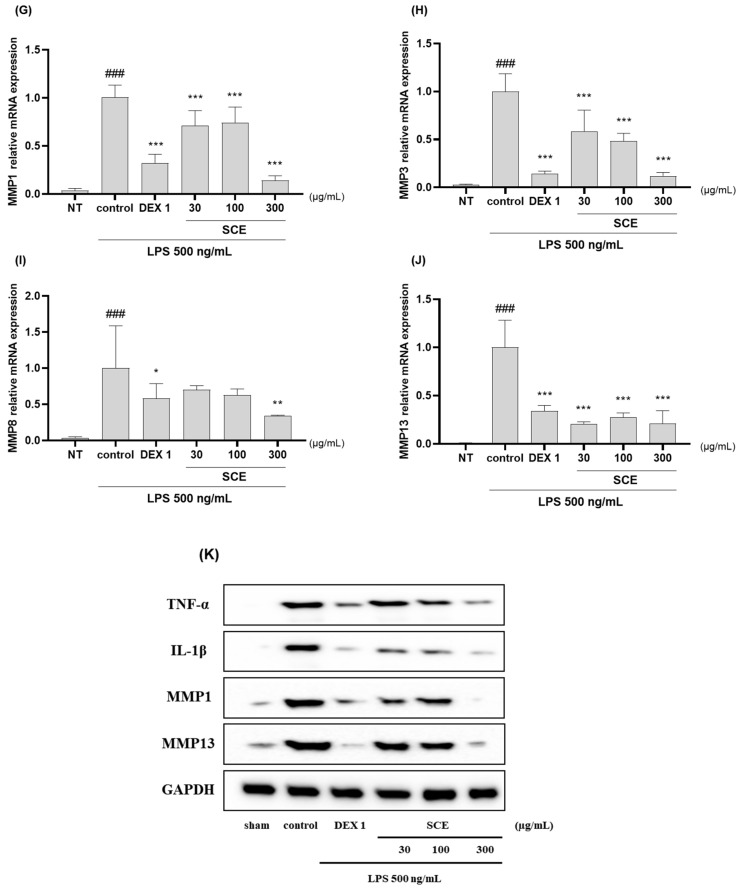
Effects of SCE on (**A**) Mitochondria Reduction Activity (MRA) and (**B**) LPS-activated NO generation. qRT-PCR analysis of (**C**–**J**) TNF-α, PGE2, IL-6, IL-1β, MMP-1, -3, -8, and -13 mRNA expression (Table 2) in LPS-activated RAW264.7 cells. Cells were incubated with 1 µg/mL of dexamethasone and 30–300 µg/mL of SCE and then treated with LPS during 24 h. Protein expression analysis of (**K**) TNF-α, IL-1β, MMP-1, and MMP-3 in RAW264.7 cells. Cells were treated with 30–300 µg/mL of SCE and 500 ng/mL of LPS for 24 h. * *p* < 0.01 vs. control, ** *p* < 0.05 vs. control, *** *p* < 0.001 vs. control, and ### *p* < 0.001 vs. sham by one-way ANOVA and Dunnett’s test. NT: non-treated, DEX 1: 1 μg/mL of dexamethasone.

In LPS-activated RAW264.7 macrophages, the anti-inflammatory effects of SCE were investigated. SCE inhibited inflammation by reducing NO concentrations and the protein expression of TNF-α, IL-1β, MMP-1, and MMP-3. In RAW264.7. No potential cytotoxicity of SCE was observed at concentrations up to 300 g/mL (Figure 7A). SCE also inhibited LPS-induced NO production (Figure 7B). As shown in Figure 7C–J, the mRNA expression of TNF-α, PGE2, IL-6, IL-1β, MMP-1, -3, -8, and -13 significantly increased in LPS-activated cells. Inflammatory cytokines and cell mediators were inhibited by 30–300 µg/mL SCE and 1 g/mL dexamethasone. The anti-inflammatory effect of SCE was dose-dependent, and 300 µg/mL of SCE showed similar levels of effects with dexamethasone. Western blotting was performed to assess the anti-inflammatory effects of SCE in LPS-activated RAW264.7 cells. SCE treatment inhibited the protein expression TNF-α, IL-1β, MMP-1, and MMP-3 such as mediators and pro-inflammatory cytokines in LPS-activated RAW264 cells (Figure 7K).

## 4. Discussion

The results of the above study showed that SCE could inhibit pain and cartilage destruction, the diverse pathological findings of OA, and that this effect was dose dependent. In addition, SCE exhibited pronounced anti-inflammatory effects based on its bioactivity against a number of key therapeutic targets, including TNF-α, IL-1β, MMP-1, MMP-3, and MMP-13. These findings were validated using both in vivo and in vitro models. Notably, to the best of our knowledge, this is the first report of the potential OA-mitigating effects of the stem of *Sorbus commixta* Hedl.

Two phenotypes not directly related to the most important therapeutic targets are persistent pain and irreversible cartilage destruction [36]. In recent years, chronic uncontrolled synovial inflammation has been suggested as an important contributor to progressive joint damage and noxious stimuli in OA [37]. Thus, many DMOAD candidates under investigation focus on preventing disease progression by inhibiting the inflammation-based multipathology of OA [38]. In the in vivo experiments conducted in this study, SCE was tested in a rat model of MIA-induced OA and showed statistically significant analgesic effects. Moreover, a high dose of SCE (300 mg/kg) was equivalent to the active control indomethacin. The analgesic activity of SCE was cross-validated in an acetic-acid-induced writhing model, and its effect was comparable to that of ibuprofen. SCE also has a marked suppressive effect on cartilage destruction. The results of the present study suggest that SCE has the potential to modulate two important phenotypes of OA pathophysiology and is a promising drug candidate worthy of further investigation.

In the present study, we used both in vivo and in vitro models to explore the mechanisms underlying the effects of SCE. The results showed that SCE exhibited pronounced anti-inflammatory activity against multiple potential targets, consistent with the primary goal of DMOADs, inhibiting low-grade inflammation in OA [39]. This inference is supported primarily by the results of the present study, in which the proinflammatory cytokines IL-1β and TNF-α in the blood of MIA rats were suppressed after SCE administration to a similar or greater extent than indomethacin. IL-1β is an important pro-inflammatory cytokine that is directly involved in chronic low grade inflammation in OA [40]. Besides contributing to the production of nerve growth factors mediating pain development, IL-1β induces progressive joint destruction by promoting matrix metalloproteinase production in chondrocytes and osteoclast differentiation [41,42]. Furthermore, TNF-α is a major pro-inflammatory cytokine that is involved in the inflammatory pathology of early-onset OA and is an important target for anti-inflammatory therapy [43]. This cytokine is directly involved in triggering pain in OA by acting on nociceptive sensory neurons or inducing pain mediators such as prostaglandins [44]. A study reported that pain severity, as measured by the WOMAC, correlated with TNF-α [45]. The previous characterization of these two major pro-inflammatory cytokines and the dose-dependent findings of analgesia, inhibition of chondrolysis, and anti-inflammation of SCE in this study are consistent. Based on these findings, the alleviating effects of SCE on OA may be explained by its potent anti-inflammatory mechanism.

The activity of SCE, which encompasses the above effects, was further confirmed by in vitro studies on a number of cytokines. For all cytokines tested, a statistically significant inhibition of mRNA expression was observed in response to SCE administration in a discernible dose-dependent manner. The same trend was observed for protein expression, particularly for IL-6, MMP-1, MMP-3, and MMP-13, in addition to TNF-α and IL-1β, for which serum levels have already been documented. IL-6 is considered an important therapeutic target in the development of DMOADs, and high levels of this cytokine correlate with an increased incidence of knee OA and severe cartilage loss over time [39]. A previous study using a mouse model reported that systemic inhibition of IL-6 may attenuate OA by inhibiting chondrocyte catabolic pathology via Stat 3 signaling [46]. Meanwhile, OA increases the production of several matrix-remodeling enzymes, including MMPs, which exacerbate joint destruction by degrading proteoglycans, collagens, and the extracellular matrix [47]. Overexpression of MMPs is considered an important therapeutic target, as it leads to the dysregulation of tissue remodeling and is involved in the pathogenesis of OA, encephalomyelitis, rheumatoid arthritis, and Alzheimer’s disease.

In the present study, we identified caffeic acid as the major active constituent of SCE. Previous experimental studies on the effects of caffeic acid on OA reported an inhibition of collagen II degradation and a reduction of inflammatory mediators, such as iNOS, COX2, MMPs, and ADAMTS5, through inhibition of IL-1β activity and suggested that caffeic acid may be involved in NF-κB and MAPK-related JNK signaling pathways [48]. Meanwhile, other preclinical studies have confirmed that caffeic acid phenethyl ester can protect cartilage and delay disease deterioration in OA models based on its inhibitory activity against NOS, COX2, PGE2, MMP3, and MMP13, with NF-κB being the main signaling pathway involved [49]. All previous studies on the efficacy of caffeic acid in alleviating OA showed strong consistency with the results of the present study. This suggests that the effect of SCE in this study was largely correlated with that of caffeic acid. Further research is needed to determine whether caffeic acid can be used as a marker to reflect the efficacy of SCE. It is worth noting that the main mechanism of action of caffeic acid involves the NF-κB signaling pathway. The fact that this pathway is highly correlated with TNF-α, IL-1β, various MMPs, PGE2, NOS, and COX2 in OA pathology, as observed in this study, suggests that NF-κB signaling and tumors may be the primary therapeutic target path [50]. Specifically in the case of OA, NF-κB signaling is known to promote the release of MMPs, such as MMP-1, MMP-3, and MMP-13, and subsequent positive feedback leads to articular cartilage degradation [51]. In addition, MMP-3 may play a role in the pathogenesis of OA by exacerbating inflammation, promoting angiogenesis, and accelerating cartilage degradation. MMP-13 is the major collagenase in OA, so its inhibitors are under vigorous research [47,52]. These studies consistently support the hypothesis that SCE exerts analgesic and chondroprotective effects owing to its broad anti-inflammatory activity against multiple targets in OA. way for SCE in OA. Therefore, it is worth conducting further studies to verify this hypothesis.

## 5. Conclusions

Based on these findings, it can be concluded that SCE exerts osteoarthritic effects through its anti-inflammatory activity. This study found that SCE had a statistically significant and dose-dependent analgesic and inhibitory effect on articular cartilage degradation. Meanwhile, the anti-inflammatory activity of SCE was confirmed by its suppressive effects on various inflammatory markers, including TNF-α and IL-1β which are directly related to OA, which were consistent in both in vivo and in vitro models. However, the key pathways and multi-target mechanisms underlying these effects have not been evaluated in this study. Furthermore, the anti-inflammatory and chondroprotective effects observed in this study may be related to the antioxidant properties of SCE, as they were exerted by targeting IL-1β and TNF-α. Further experiments on the effect of inhibiting the production of reactive oxygen species are needed to confirm this further. In this regard, follow-up studies on the mechanisms of anti-OA effects of SCE would be worthwhile. If the above hypothesis is clearly demonstrated in subsequent studies, the potential of SCE as a DMOAD candidate will become clearer.

## Figures and Tables

**Table 1:** mRNA primer sequence for OA-induced cartilage tissues.

Gene Name	Primer Sequence (5′ → 3′)	
**TNF-α**	F	GCATGATCCGAGATGTGGAA
	R	GATGAGAGGGAGCCCATTTG
**COX-2**	F	GTTCCAACCCATGTCAAAAC
	R	TGTCAGGAATCTCGGCGTAG
**IL-6**	F	TCCGCAAGAGACTTCCAGC
	R	CCTCCGACTTGTGAAGTGG
**IL-1β**	F	AACTCAACTGTGAAATAGCAGC
	R	TCCACAGCCACAATGAGTG
**MMP-1**	F	AACTTGGGTGAAGACGTCCA
	R	TCCTGTCACTTTCAGCCCAA
**MMP-3**	F	GTACGGCTGTGTGCTCATCC
	R	TCAGCCCAAGGAACTTCTGC
**MMP-8**	F	TCTGTTCTTCTTCCACACACAG
	R	GCAATCATAGTGGCATTCCT
**MMP-13**	F	ACCTTCTTCTTGTTGAGTTGGA
	R	CTGCATTTCTCGGAGTCTA
**NOS2**	F	AGTCAACTACAAGCCCCACG
	R	GCAGCTTGTCCAGGGATTCT
**PGE2**	F	TGTGTGTACTGTCCGTCTGC
	R	CAGGGATCCAGTCTCGGTGT
**GAPDH**	F	CTTGTGACAAAGTGGACATTGTT
	R	TGACCAGCTTCCCATTCTC

TNF-α: tumor necrosis factor-alpha, COX-2: cyclooxygenase-2, IL-6: interleukin-6, IL-1β: interleukin 1 beta, MMP: matrix metalloproteinase, NOS: nitric oxide synthase, PGE2: prostaglandin E2, GAPDH: glyceraldehyde 3-phosphate dehydrogenase.

**Table 2:** mRNA primer sequence for LPS-activated RAW264.7 cells.

Gene Name	Primer Sequence (5′ → 3′)	
**TNF-α**	F	GAGAAGTTCCCAAATGGCCT
	R	AGCCACTCCAGCTGCTCCT
**PGE2**	F	CTGGTAACGGAATTGGTGC
	R	TGGCCAGACTAAAGAAGGTC
**IL-6**	F	CACTTCACAAGTCGGAGGCT
	R	CAAGTGCATCATCGTTGTTC
**IL-1β**	F	CCAGCTTCAAATCTCGCAGC
	R	GTGCTCATGTCCTCATCCTGG
**MMP-1**	F	ATGCCTAGCCTTCCTTTGCT
	R	TTCCAGGTATTTCCAGACTG
**MMP-3**	F	AAGTTCCTCGGGTTGGAGAT
	R	ACCAACATCAGGAACACCAC
**MMP-8**	F	CAATCAATTCCGGTCTTCGA
	R	GGTTAGCAAGAAATCACCAGA
**MMP-13**	F	AACCAAGATGTGGAGTGCCT
	R	GACCAGACCTTGAAGGCTTT
**GAPDH**	F	ATGGTGAAGGTCGGTGTG
	R	GCCGTGAGTGGAGTCATAC

TNF-α: tumor necrosis factor-alpha, PGE2: prostaglandin E2, IL-6: interleukin-6, IL-1β: interleukin 1 beta, MMP: matrix metalloproteinase, NOS: nitric oxide synthase, GAPDH: glyceraldehyde 3-phosphate dehydrogenase.

## Abbreviations

DEX: dexamethasone, DMOADs: disease-modifying osteoarthritis drugs, LPS: lipopoly saccharide, MIA: monosodium iodoacetate, MMP: matrix metalloproteinase, NSAIDs: nonsteroidal anti-inflammatory drugs, NO: Nitric Oxide OA: osteoarthritis, PVDF: polyvinylidene fluoride, RT: room temperature, SCE: *Sorbus commixta* Hedl. stem extracts.
